# Virtual Reality Meets Non-invasive Brain Stimulation: Integrating Two Methods for Cognitive Rehabilitation of Mild Cognitive Impairment

**DOI:** 10.3389/fneur.2020.566731

**Published:** 2020-09-30

**Authors:** Valentina Mancuso, Chiara Stramba-Badiale, Silvia Cavedoni, Elisa Pedroli, Pietro Cipresso, Giuseppe Riva

**Affiliations:** ^1^Applied Technology for Neuro-Psychology Lab, Istituto Auxologico Italiano, Istituto di Ricovero e Cura a Carattere Scientifico, Milan, Italy; ^2^Department of Psychology, E-Campus University, Novedrate, Italy; ^3^Department of Psychology, Catholic University of the Sacred Heart, Milan, Italy

**Keywords:** virtual reality, transcranial magnetic stimulation, mild cognitive impairment, cognitive rehabilitation, cave, dorsolateral prefrontal cortex, non-invasive brain stimulation, executive functions

## Abstract

Mild cognitive impairment (MCI) refers to a subtle, general cognitive decline with a detrimental impact on elderlies' independent living and quality of life. Without a timely diagnosis, this condition can evolve into dementia over time, hence the crucial need for early detection, prevention, and rehabilitation. For this purpose, current neuropsychological interventions have been integrated with (i) virtual reality, which immerses the user in a controlled, ecological, and safe environment (so far, both virtual reality-based cognitive and motor rehabilitation have revealed promising positive outcomes); and (ii) non-invasive brain stimulation, i.e., transcranial magnetic or electric brain stimulation, which has emerged as a promising cognitive treatment for MCI and Alzheimer's dementia. To date, these two methods have been employed separately; only a few studies (limited to motor rehabilitation) have suggested their integration. The present paper suggests to extend this integration to cognitive rehabilitation as well as to provide a multimodal stimulation that could enhance cognitive training, resulting in a more efficient rehabilitation.

## Introduction

To a certain degree, cognitive decline is a physiological change occurring during the aging process that occasionally evolves into a subtle condition known as mild cognitive impairment (MCI) (1). Despite being undiagnosable as proper dementia, at least following a categorical approach, this condition can have a detrimental impact on elderlies' cognitive functioning and worsen their conditions over time, even up to a point where an elderly presents with a frank dementia. However, MCI is also likely to either revert back to normal cognition or stabilize over time (2).

Both MCI patients and their caregivers frequently report concerns about worsening cognition in areas such as everyday memory, language, visuospatial skills, planning, organization, and divided attention (3). The decline in cognitive functioning negatively affects elderlies' independent living and their ability to safely and autonomously carry out instrumental activities of daily life (IADLs), an assessment instrument that measures an individual's ability to perform daily activities (4) such as grocery shopping, managing medications and/or money, and housework. In fact, MCI individuals are less able to perform IADLs than their healthy counterparts (5), with detrimental effects on their wellbeing (3, 6) and an increased risk of developing dementia (7). Since activity restriction underlies the expression of cognitive impairment in daily life, IADLs might enable the detection of early deficits experienced during daily activities beyond those captured by neuropsychological tests (8).

MCI is most commonly referred to as a degenerative etiology (i.e., Alzheimer's disease [AD], frontotemporal dementia, dementia with Lewy bodies), but vascular (i.e., vascular cognitive impairment), psychiatric (e.g., depression), genetic (APOE and TOMM40 genes) and other medical conditions (e.g., uncompensated heart failure, poorly controlled diabetes mellitus, or chronic obstructive pulmonary disease) can also contribute to the determination of cognitive impairment (9, 10). Clinicians classify MCI into broadly differentiated subtypes—amnestic (aMCI) and non-amnestic (naMCI)—based on whether the condition impairs one or multiple cognitive domains (1). aMCI refers to patients who exhibit episodic memory impairments as confirmed by neuropsychological tests and is associated with higher risk of further conversion to AD (11, 12). naMCI refers to patients with neuropsychological deficits in non-memory cognitive domains (12).

Neurobiological studies have revealed that cognitive impairment affecting memory (e.g., episodic memory) and other domains (e.g., executive control, language, or visuospatial abilities) is associated with altered neural activity in prodromal AD (i.e., aMCI): the entorhinal cortex and hippocampus are first affected by histopathological changes, followed by the parahippocampal gyrus, the temporal pole, and the inferior and middle temporal gyri (9, 12–16). While primary cortices seem to be less vulnerable to deterioration, associative areas are the most compromised: among them, the prefrontal cortex (PFC) shows a higher decline (17). Discriminating between normal and pathological neural changes is crucial in order to formulate an accurate diagnosis and a prompt treatment plan (18).

The conversion to dementia usually occurs within 3 years after the diagnosis of MCI, and this rate critically drops in the following years (19). Therefore, a delayed intervention could be ineffective when the cognitive decline is close to the dementia stage (20). Furthermore, the timing of the intervention also affects cost-effectiveness: therefore intervening 2 years prior to standard diagnosis would allow the maximum net benefit of the disease-modifying intervention (19).

Thus, this long “intermediate” phase provides a critical opportunity for therapeutic intervention. Cognitive interventions for MCI usually encompass a variety of approaches, heterogeneous in terms of methods and contents. Among them, cognitive training could be considered as a secondary prevention method, particularly for “at risk” groups. It generally consists of theoretically driven skills and strategies which guide and encourage patients to perform tasks engaging several cognitive domains (21). Previous studies have showed that, on one hand, MCI patients show impairments in everyday memory, language, visuospatial skills, planning, organization, and divided attention affecting daily activities, as also confirmed by worse scores in IADL (7, 12); on the other hand, MCI patients could exhibit different neural impairments, as previously mentioned (12).

Considering that MCI is characterized by both cognitive-behavioral and neural impairments, a successful rehabilitation process should address both of them and could benefit from the integration of technological advancements. A plausible candidate could be virtual reality (VR) due to its psychological and technological features: VR scenarios simulate daily life situations in which the user can feel immersed and interact with an environment updated in real-time, while also receiving dynamic multisensory feedback (21–25). A recent systematic meta-analysis showed that specific VR environments built on principles of neurorehabilitation that potentially enhance learning and recovery seem more efficacious than non-specific VR-based treatments or conventional therapies (26). Non-invasive brain stimulation (NIBS) including transcranial magnetic stimulation (TMS) has been showed to be efficacious for cognitive rehabilitation as well (27).

The widespread of neurodegenerative diseases have increased the demand for the development of new techniques to support the rehabilitation. Since the recovery is complex, there is a growing interest in the development of new technologies for improving outcomes of conventional clinical intervention strategies. The aim of the present paper is two-fold: first, to provide a brief review of current evidence regarding the benefits of non-invasive technologies (VR and TMS) on MCI cognitive rehabilitation. Second, to propose an integrated intervention approach consisting of VR-based cognitive training and neural stimulation by means of TMS. The integration of existing technologies does not replace MCI's standard rehabilitative methods, but rather upgrades them in order to create a novel approach that guarantees an ecological setting and takes action on different aspects of this clinical entity, thereby fostering cognitive improvements. Therefore, the present paper aims to propose a new integrated, multimethod approach acting on both a neural-cognitive and behavioral-cognitive level for MCI by means of VR and TMS.

## A New Integrated Approach

This section will be structured as follows: (i) the features of two existing interventions for MCI (i.e., VR and TMS) will be summarized, as well as (ii) the recent literature about their integration in motor rehabilitation of MCI and other clinical applications; (iii) finally, a discussion of a novel approach integrating these methods for MCI cognitive rehabilitation.

### Virtual Reality in Rehabilitation

Available methods for MCI rehabilitation consist of cognitive stimulation or cognitive training, usually in the paper-and-pencil format and conducted in an isolated and non-ecological setting (28), consisting, for example, of exercises of categorization, semantic association, classification and mental imagery according to specific goals (memory for proper names, object location, etc.) (28). Recently, new technologies have been increasingly implemented in clinical settings: VR is an immersive technology using 3D computer generated environments. When a user is immersed in virtual reality, he/she experiences the sense of “being there” inside the virtual environment while knowing for sure that he/she is not (29, 30); this allows to recreate lifelike contexts in an ecological, safe and controlled setting (31–34). The sense of presence can be considered as a neuropsychological phenomenon resulting from our biological inheritance and our experience as active agents in our surrounding environment. Fully immersive VR scenarios create a strong sense of Place Illusion and Plausibility Illusion for the user, and result in realistic emotional reactions to the situations encountered in VR, perceptual accuracy, and a strong sense of agency and control over the virtual environment (34–36). These crucial features have fostered the widespread employment of VR in clinical rehabilitation (22, 25, 37, 38). Depending on the degree of immersiveness, VR devices can fall into three categories: non-immersive (e.g., user interacts with the environment with a keyboard and mouse); semi-immersive (e.g., user usually stands in front of a large screen, and gesture and location can be tracked); and fully-immersive [e.g., user wears a head-mounted display (HMD) that involves the entire vision or is immersed in the cave automatic virtual environment (CAVE), a four-walled virtual environment that provides a stronger sense of presence). VR also allows users to interact with virtual objects and to receive multisensory feedback (e.g., visual, auditory, kinesthetic) corresponding to that received in real life through the sensory system (39, 40). Synchronization of the different stimuli corresponding to different sensory streams allows the user to experience the virtual environment as realistic and results in realistic behaviors of users experiencing place illusion (41). Indeed, place illusion is defined as “the illusion of being in a place in spite of the sure knowledge that you are not there” and it differs from the plausibility illusion which is defined as “the illusion that what is apparently happening is really happening, in spite of the sure knowledge that it is not” (42, 43). The behavioral correlate of these illusions is that the user behaves in the virtual environments as he would do in the real world (44). This important feature of VR is what distinguishes it from all other types of media. Moreover, VR allows personalized therapies in a controlled way by modulating difficulty level, environments (e.g., adding or removing cues) and modality of interaction tailored to the patient's needs. The possibility of creating safe, ecological, and standardized settings has supported the employment of VR in neurorehabilitation because it allows cognitive trainings that are relevant for real contexts (22, 45, 46), supported by its potential to promote neuroplasticity (47–50). With respect to MCI, various studies have showed VR's potential to enhance cognitive functions [for reviews, see (36, 38)]. For instance, Optale and colleagues (51) showed that 36 sessions of VR-based memory training in a fully immersive environment (provided by an HMD and enriched by visual and auditory stimuli) improved patients' post-treatment Mini Mental State Examination (MMSE) scores compared to the control group—receiving musical therapy intervention—whose MMSE scores decreased instead. Moreover, memory showed improvements as well, as assessed by digit span forward and verbal story recall (51). Similar promising results were observed in an MCI sample after non-immersive VR sessions consisting of performing tasks and navigating a virtual home and supermarket (52). Patients exhibited significant improvements on the Montreal Cognitive Assessment (MoCA) and IADL after a 3-week VR-based cognitive and physical training: interestingly, after the VR intervention, functional near-infrared spectroscopy (NIRS) revealed decreased brain activation of the prefrontal areas as a result of increased neural efficiency during the training (53). Overall, several studies have highlighted the potential of VR in memory (54–57) and executive functions rehabilitation (58–61). In particular, it has been shown that the multisensory stimulation has a positive impact on both the sense of presence and memory functioning (62). At the same time, by creating complex and ecological environments, VR provides the possibility to train different executive functions (e.g., visual attention, planning, problem solving) along with motor demands, enhancing cognitive functions in daily living (59).

In fact, according to the compensatory model, demented brains show broader activation as a compensatory strategy to preserve intact cognitive functions (63, 64). Therefore, this study expands previous literature about VR efficacy to improve neural efficiency in prefrontal areas (53). Besides cognitive enhancement, VR treatments have beneficial psychological effects: participants reported feeling more enthusiastic, relaxed, energetic and, most importantly, less worried, stressed, and anxious (65). Despite these promising results, however, the heterogeneity of the studies, in terms of VR devices' different degrees of immersivity and the variety of protocols, makes it difficult to clarify the mechanisms underlying VR's effectiveness. A recent meta-analysis (66) suggests three plausible mechanisms. (i) Enjoyment: VR provides fun and engaging experiences with different tasks (e.g., exploration, challenges) that motivate patients to complete them. Conversely, patients perceive conventional rehabilitation methods, consisting of repeated behaviors without immediate feedback, as repetitive and boring. (ii) Physical fidelity: VR offers realistic scenarios that allow users to perform and practice behaviors resembling daily activities, whereas traditional rehabilitation programs focus on non-familiar behaviors. (iii) Cognitive fidelity: VR environments can be built according to specific cognitive tasks and the cognitive load required by the transfer environment. On the contrary, conventional rehabilitation is set in a relatively stimulus-free environment with limited cognitive fidelity.

### Transcranial Magnetic Stimulation (TMS) in Rehabilitation

TMS is a form of non-invasive brain stimulation that induces an electrical field through a coil placed on the surface of the scalp over a targeted stimulation site (67). Depending on selected parameters (i.e., frequency, intensity, number of pulses delivered, type of coil, and location of the stimulation), TMS pulses can either excite or inhibit cortical activity and induce long- or short-term neural and behavioral changes (68). Depending on the number of pulses delivered, TMS can be single-pulse (i.e., when only one stimulus at a time is employed), paired-pulse (i.e., when pairs of stimuli separated by an interval are delivered) or repetitive (rTMS) in case of trains of stimuli (69).

TMS has increasingly been applied in several research and clinical fields (70), including in cognitive rehabilitation of MCI and dementia (27, 71–73). One study (27) reviewed the potential of TMS in modulating cognitive functions both in MCI and AD: the majority of the studies employed multiple sessions of high-frequency (>5 Hz) rTMS over the dorsolateral prefrontal cortex (DLPFC). Overall, TMS appeared effective at significantly improving memory and executive functions. Another study (72) reported long-term memory and executive functioning improvements after 10 high-frequency rTMS sessions over the left DLPFC. One study (74) considered the effectiveness of rTMS over the DLPCF at reducing apathy, a symptom frequently reported in MCI patients: a significant improvement in apathy scores resulted following 10 sessions of active TMS compared to sham. Interestingly, authors observed positive outcomes in executive functions as well, as assessed by the Trail Making Test (75). Cognitive benefits resulting from TMS interventions can be explained by the reorganization of the brain networks following the induced changes in cortical excitability. In other words, high-frequency rTMS sessions may determine an improvement in terms of synaptic plasticity, with implications for reorganization of cognitive domains (76, 77).

However, the mechanisms underlying TMS effects are still unclear. One hypothesis is that high-frequency TMS induces intracortical inhibition. In other words, discharging an electrical field causes gamma-aminobutyric acid (GABA) levels to increase, suppressing the activity (78). This temporary neuro-disruption, called a “virtual lesion,” causes a disruption of perceptual, motor, and cognitive processes in the human brain (79). Another hypothesis is that TMS might determine a random neural noise by amplifying the background activity (80). Other authors suggested that TMS could disrupt the temporal relation between neurons implicated in a more extended circuit activated by the task (81). Overall, the effects of TMS are heterogeneous and seemingly dose- and context-dependent. On one hand, the effectiveness of TMS depends on the frequency and duration of the stimulation: its effects are more pronounced as long as both TMS trains and frequency increase. On the other hand, the effects depend on the level of cortical excitability at the moment of the stimulation: the pulse recruits as many neurons are close to the firing threshold (76). Overall, the effectiveness of TMS remains hindered by a number of methodological challenges, including a lack of clear consensus about the optimal stimulation parameters, with variability in the type, frequency, intensity, location, and duration of stimulation.

### Virtual Reality and Non-invasive Brain Stimulation

The joint application of NIBS and VR has been previously investigated in different clinical settings to improve the clinical outcomes of conventional therapies. VR provides a controlled, ecological, and appealing setting that could be personalized according to the patient needs, whereas NIBS might alter the neurophysiology underpinning cognitive functions. For this purpose, different studies have suggested that the combination of these technologies could be more synergic than stand-alone treatments. For instance, it has been employed to induce embodiment for an artificial hand (82), to treat spider phobia (83) and in interventions in different populations, such as children with cerebral palsy, post-stroke patients, and healthy people (84). In rehabilitation settings, different studies have investigated the potential of joining VR and transcranial direct current stimulation (tDCS)/TMS for the rehabilitation of the upper limb, one of the most common deficits following a stroke (84–89). Kim and colleagues (90) found that VR wrist exercise after tDCS had greater immediate and sustained corticospinal facilitation effects than exercise without tDCS and tDCS without exercise. Furthermore, this corticospinal facilitation lasted for 20 min after the exercise in the VR+tDCS condition compared to the control groups. Recently, a meta-analysis (88) proved the effectiveness and suitability of NIBS-VR integration for motor rehabilitation of the upper limb. While different studies have proved the efficacy of the joint application of NIBS and VR for motor rehabilitation, to our best knowledge, no studies have investigated the same approach for cognitive rehabilitation.

### Virtual Reality and Transcranial Magnetic Stimulation for Cognitive Rehabilitation

This section discusses an integrated intervention approach that encompasses both TMS and a training VR for cognitive rehabilitation of MCI.

**Figure 1:**
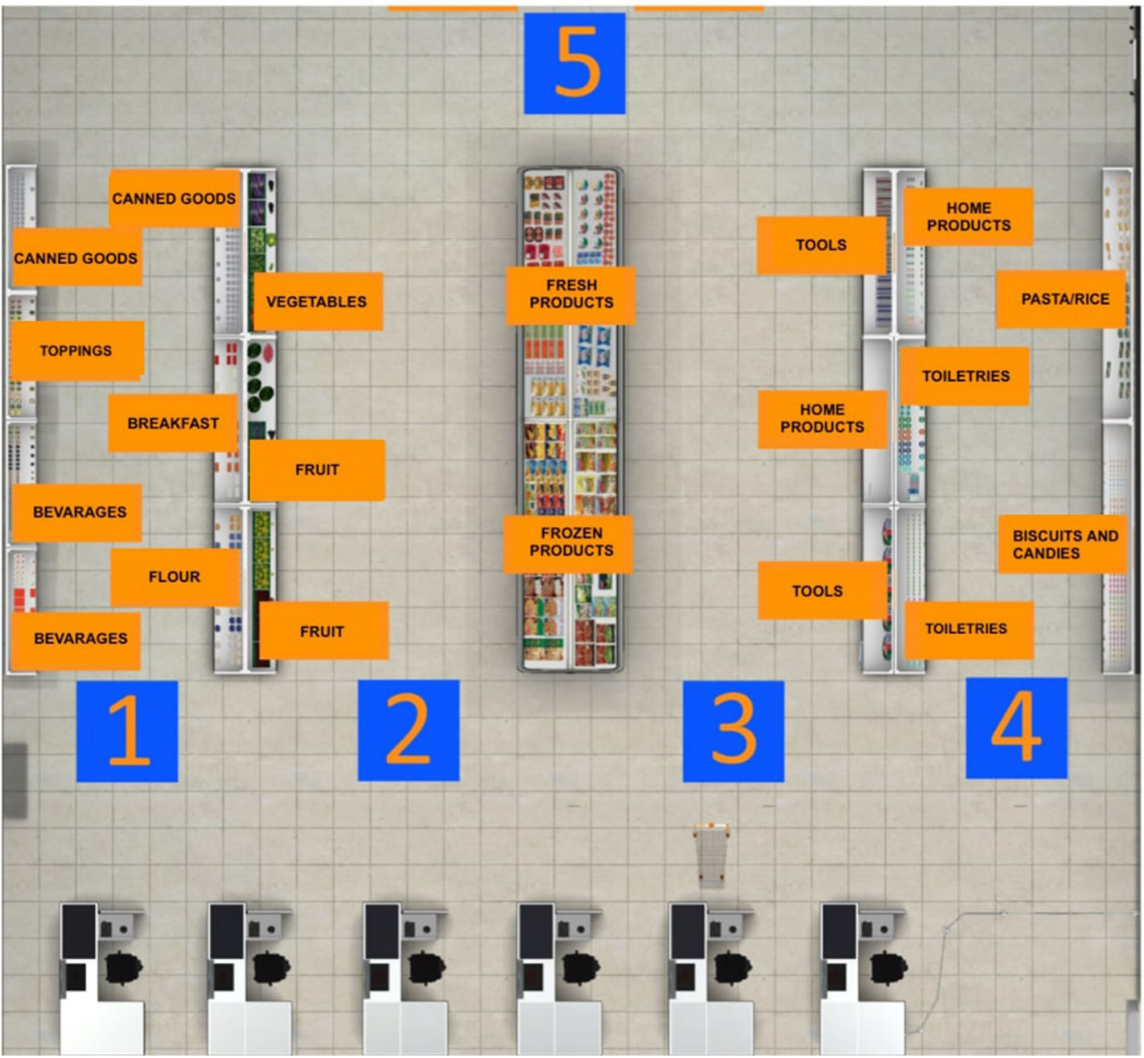
Virtual supermarket task map. Before starting the navigation into the virtual supermarket, this map is projected on one of the four walls of the CAVE. The patient has to memorize the position of every category of products he/she has to buy.

**Figure 2:**
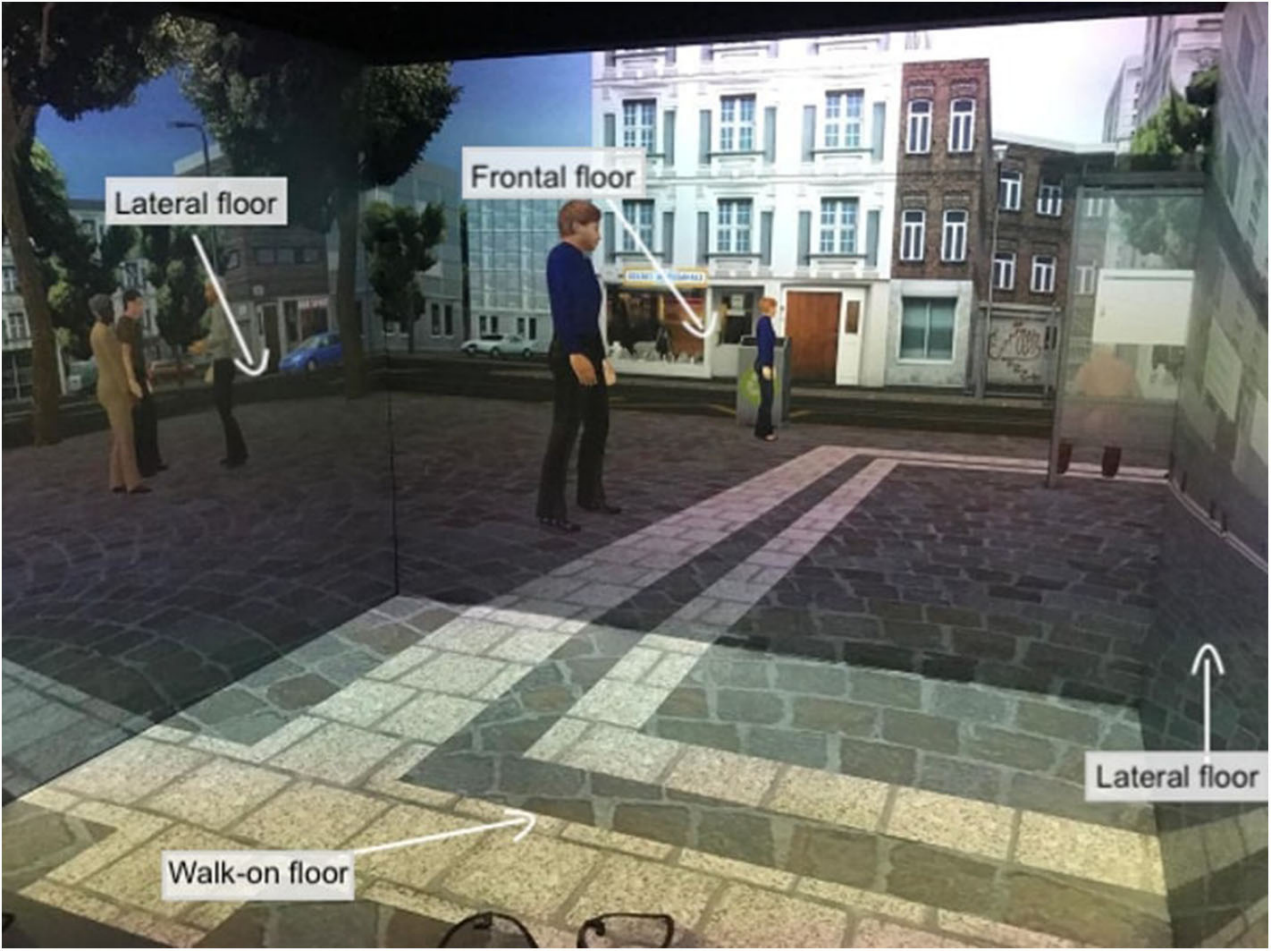
Virtual city task. This figure shows the patient's point of view when immersed in the 3-D CAVE. The projections on the four floors move synchronously with the user's navigation through the controller.

VR interventions showed positive outcomes in cognitive and motor functioning in patients with MCI or dementia, as reported by a recent meta-analysis (46). Despite the mechanisms underlying the application of TMS for cognitive rehabilitation being uncertain, heterogeneous, and ambivalent, studies targeting cognitive rehabilitation suggested that aMCI and AD patients benefited from its employment (27). Therefore, a plausible hypothesis is that high-frequency rTMS over the left DLPFC might recruit more neural resources from the prefrontal cortex by inducing an electrophysiologically excitatory effect. This stimulation could also enhance the efficiency of resources to deploy for conflict resolution during multiple stages of cognitive control processing. In other words, rTMS could induce a greater activation and efficacy of the prefrontal cortex (91), an area that is involved in accomplishing the VR tasks. In fact, an eclectic approach to cognitive rehabilitation achieves greater improvements based on the assumption that cognitive deficits are also determined and influenced by physical (e.g., illness, blood pressure, pain, sleep), emotional (e.g., anxiety, annoyances, arousal, mood), social (e.g., relationships, status, social pressure) and motivational (e.g., distractibility, goals, incentives) aspects (92). Specifically, a plausible integrated intervention could include 10 training sessions of 40 min, composed of rTMS (active or sham) and the virtual-based training. Before the first session and at the end of the tenth, aMCI patients will receive a neuropsychological assessment. First, high-frequency rTMS will be delivered over the left DLPCF, a region known to be involved in executive functions and in long-term memory due to its interaction with the medial-temporal network, including the hippocampus (93–95). After each session of rTMS, patients wearing 3-D glasses will be immersed inside a CAVE^1^, a virtual room-sized cube, at I.R.C.C.S. Istituto Auxologico Italiano (Milan, Italy), in which they will be exposed to two different environments (96). Patients will be first immersed in a virtual supermarket (Figure 1) in which they would be able to move around thanks to an Xbox controller. Tasks will consist of selecting different products on shelves according to precise rules, with increasing difficulty. Every task, according to rules and goals, will require both executive (e.g., planning, problem solving, and divided attention) and memory functioning (e.g., remembering rules). Patients will be then immersed in a virtual city (Figure 2) in which they will be required to perform two tasks. At the beginning, they will be placed in the center of a square and asked to move around in the virtual city, looking for a target object previously identified with the therapist. Then, they will be placed in a random location in the city and asked to retrieve the position of that object. This city task will aim to enhance spatial memory, navigation, and planning strategies.

The neuropsychological assessment will target general cognitive functioning through Addenbrooke's Cognitive Examination (ACE-R) (97) and MoCA (98). Executive functioning (planning, initiating, and monitoring) will be assessed with the Trail Making Test (TMT version A and B) (75) and the Stroop test (99). Memory abilities will be evaluated by Digit Span (100) and Babcock (101). Visuo-spatial abilities will be evaluated by Tower of London (ToL) (102). Mood will be assessed by the State-Trait Anxiety Inventory (STAI) (103) and Geriatric Depression Scale (GDS) (104). Lastly, activities of daily living (ADL) (105) and IADL (4) will be collected.

During the entire intervention, physiological measures (e.g., heart rate variability, skin conductance) will be collected.

## Discussion and Conclusion

MCI is a transitional subclinical entity creating a fine line between normal and pathological aging. Thus, early interventions are essential to preserving cognitive functioning and, as far as possible, to decelerating its evolution toward dementia. MCI may benefit from an efficacious intervention deeming that the brain might still be able to compensate for its deficiencies and to support the acquisition and retention of the impaired cognitive functions. With the progression of pathological conditions and the spreading of lesions instead, the brain might no longer be able to compensate (106, 107). Thus, a prompt rehabilitation might be helpful in delaying the progression to dementia. Considering that both VR-based training and neuromodulation capitalize on neuroplasticity, they can enhance the therapeutic mechanisms in a complementary way. On one hand, rTMS aims to increase excitability within the lesioned hemisphere and to suppress stimulation to the contralesional hemisphere, namely, reducing inter-hemispheric inhibition from the contralesional side (108–111). Specifically in aMCI patients, the DLPFC is characterized by abnormal functional connectivity, determining several cognitive and emotional impairments (112). Stimulation of the prefrontal cortex is expected to enhance activation and efficiency in this area responsible for both executive functions (e.g., working memory and flexibility) and long-term memory due to its connection with the medial-temporal network (e.g., the hippocampus) (93–95). On the other hand, VR-based intervention will provide patients with lifelike functional tasks (like doing groceries and walking around the city) that involve cognitive domains, physical activity, and emotional–behavioral aspects. Given the potential of VR to provide an ecological and immersive setting, along with immediate feedback, the repetitive practice of these functional tasks would facilitate a complex cognitive processing strengthened by enjoyment and attractiveness, which might facilitate motivation and engagement. Patients would be required to tap into their attentional, mnemonic, planning, flexibility, and navigation abilities to accomplish the virtual tasks. It is plausible to expect that this multi-session, multi-modal intervention would facilitate the transfer of these abilities to real-life daily activities as well. Furthermore, elderlies' enjoyment could promote their engagement and treatment adherence.

The integration of VR and TMS may allow a more sensitive rehabilitation of cognitive symptoms while simultaneously modulating the impaired neural circuits to provide a stronger beneficial effect. It is plausible that the implementation of neural modulation within an ecological virtual environment may allow elderlies to benefit even more than stand-alone intervention. Besides, available interventions for MCI are frequently conducted in isolated and artificial situations, thus allowing evaluation biases.

The sense of presence, i.e., the subjective sense of being there in a virtual environment rather than in the actual physical environment, is central in a VR experience. As the other subjective feeling states, presence depends on a set of predictions about the interoceptive state of the body (113). In this regard, predictive coding theory can be used to describe the relationship between top-down prediction/expectation signals and bottom-up prediction error signals. In immersive environments, the experience of being there is based on the synchronization between expected and actual sensorimotor signals, leveraging a prediction-based model of behavior (114). In this way, immersive environments could allow to foster cognitive modeling/change, by providing realistic life-like multisensory experiences. According to it, we expect that neural and cognitive manipulation through rTMS and VR, respectively, might yield more beneficial outcomes than standard intervention both in paper-and-pencil and computer methods. Similarly to previous results, we expect that this integrated approach would determine improvements in general cognitive, memory, visuospatial, and particularly executive functioning, as well as in IADL and ADL scores for elderlies affected by MCI. In fact, both memory and executive impairments are associated with greater ADL/IADL worsening (115). VR intervention might possibly enhance complex cognitive processing as patients repetitively practiced IADL-based functional tasks. This hypothesis is in accordance with recent literature supporting the advantages of VR in improving global cognition and IADL (53).

Moreover, by collecting physiological indexes, it would be possible to record implicit measures of internal states during the whole experience, evaluating the impact of specific experiences without interfering with them (116). Indeed, biosensors are considered a reliable method for quantitative and objective measurement of the psychophysiological signals the and behavior of participants. The potential of these measures is that they provide additional information that could deepen the understanding of peculiar patterns (116).

Nevertheless, a study based on this approach is not exempt from limitations: for instance, the different stages of aMCI patients' functional levels could provide heterogeneous results. Also, some patients might not be able to complete the intervention due to dizziness or cybersickness [i.e., motion sickness including eye fatigue, nausea, headaches, and sweating (91, 92)] when immersed in virtual environments, although studies have revealed that VR is generally well-tolerated by the elderly (101). TMS could provoke discomfort and headache as well (69). Furthermore, the heterogeneity of neural impairments of MCI and the unclear beneficial effects of TMS might influence the effectiveness of this integrated approach. However, previous studies have showed promising results in the integration of neuromodulation and VR technologies for motor rehabilitation in stroke patients (85, 90). On one hand, TMS enabled shifts in cortical activity from contralesional to ipsilesional motor areas; on the other hand, VR provided repetitive, intensive, and motivating movement tasks with real-time multimodal feedback, applying motor learning principles for stroke neurorehabilitation (49, 117).

Consistent with both empirical evidence and scientific background, we thus expect that the combination of two approaches (TMS + VR) tapping into the same mechanisms will yield deeper and longer clinical outcomes in MCI patients.

## Author Contributions

VM and CS-B conceived, defined, and wrote the first draft of the perspective. GR supervised the study. All authors revised the final version of the manuscript.

## Conflict of Interest

The authors declare that the research was conducted in the absence of any commercial or financial relationships that could be construed as a potential conflict of interest.

## Footnotes

^1^The CAVE is a virtual room-sized cube in which the 3-D visualization of the virtual environments occurs thanks to the combination of four stereoscopic projectors and four screens. Two graphics workstations, mounting Nvidia Quadro K6000 GPU with dedicated Quadro Sync cards, are responsible for the projection surfaces, user tracking and functional logic. A Vicon motion tracking system with four infrared cameras allows the tracking of specific reflective markers positioned on target objects and a correct reading of the simulated spaces and distances with a 1:1 scale ratio, thus enhancing the feeling of being immersed in the virtual scene.
